# Suppression of *Physaria fendleri* SDP1 Increased Seed Oil and Hydroxy Fatty Acid Content While Maintaining Oil Biosynthesis Through Triacylglycerol Remodeling

**DOI:** 10.3389/fpls.2022.931310

**Published:** 2022-06-03

**Authors:** Abdul Azeez, Prasad Parchuri, Philip D. Bates

**Affiliations:** Institute of Biological Chemistry, Washington State University, Pullman, WA, United States

**Keywords:** *Lesquerella fendleri* L., lipase, hydroxy fatty acid, oilseed crop, Brassicaceae, SUGAR DEPENDENT 1, castor (*Ricinus communis* L.), triacylglycerol remodeling

## Abstract

*Physaria fendleri* is a burgeoning oilseed crop that accumulates the hydroxy fatty acid (HFA), lesquerolic acid, and can be a non-toxic alternative crop to castor for production of industrially valuable HFA. Recently, *P. fendleri* was proposed to utilize a unique seed oil biosynthetic pathway coined “triacylglycerol (TAG) remodeling” that utilizes a TAG lipase to remove common fatty acids from TAG allowing the subsequent incorporation of HFA after initial TAG synthesis, yet the lipase involved is unknown. SUGAR DEPENDENT 1 (SDP1) has been characterized as the dominant TAG lipase involved in TAG turnover during oilseed maturation and germination. Here, we characterized the role of a putative PfeSDP1 in both TAG turnover and TAG remodeling. *In vitro* assays confirmed that PfeSDP1 is a TAG lipase and demonstrated a preference for HFA-containing TAG species. Seed-specific RNAi knockdown of *PfeSDP1* resulted in a 12%–16% increase in seed weight and 14%–19% increase in total seed oil content with no major effect on seedling establishment. The increase in total oil content was primarily due to ~4.7% to ~14.8% increase in TAG molecular species containing two HFA (2HFA-TAG), and when combined with a smaller decrease in 1HFA-TAG content the proportion of total HFA in seed lipids increased 4%–6%. The results are consistent with PfeSDP1 involved in TAG turnover but not TAG remodeling to produce 2HFA-TAG. Interestingly, the concomitant reduction of 1HFA-TAG in PfeSDP1 knockdown lines suggests PfeSDP1 may have a role in reverse TAG remodeling during seed maturation that produces 1HFA-TAG from 2HFA-TAG. Overall, our results provide a novel strategy to enhance the total amount of industrially valuable lesquerolic acid in *P. fendleri* seeds.

## Introduction

*Physaria fendleri*, previously known as *L. fendleri*, belongs to the mustard family (*Brassicaceae*) and is an emerging industrial oilseed crop native to the southwestern United States and northern Mexico (Al-Shehbaz and O'kane, 2002; Dierig et al., 2011; Von Mark and Dierig, 2015). *Physaria fendleri* seeds contain oil with a fatty acid composition comprised of approximately 60% of the unusual hydroxylated fatty acid, lesquerolic acid (20:1-OH; (11Z,14R)-14-hydroxyeicos-11-enoic acid; Cocuron et al., 2014). Hydroxy fatty acids (HFAs) are unusual fatty acids that accumulate in the seeds of various plant species (Ohlrogge et al., 2018) and are used as feedstocks in several industries such as pharmaceuticals, cosmetics, plastics, biodegradable polyesters, and biofuels (Mutlu and Meier, 2010; Patel et al., 2016; Chen, 2017). The primary source of HFA for the industry is castor (*Ricinus communis*) seed oil, which contains 85%–90% of the HFA ricinoleic acid (18:1-OH; [9Z,12R]-12-hydroxyoctadec-9-enoic acid; Mckeon, 2016). Unfortunately, castor seeds also produce the toxic protein ricin (Chen et al., 2005), of which has greatly limited its production in the USA and several other countries. The continued development of *P. fendleri* as an oilseed crop with enhanced oil and HFA content can provide a non-toxic alternative to castor and a valuable alternative oilseed crop containing fatty acids suitable for multiple industries.

The underlying molecular mechanisms of HFA-containing oil biosynthesis have been investigated in *P. fendleri* with the ultimate goal to enhance crop development through increasing seed oil amount and HFA content. Similar to castor, *P. fendleri* synthesizes HFA by hydroxylation of oleic acid esterified to the membrane lipid phosphatidylcholine (PC) producing ricinoleic acid (18:1-OH; Bafor et al., 1991; Vandeloo et al., 1995; Reed et al., 1997; Broun et al., 1998). In castor, PC acyl editing produces ricinoleoyl-CoA which is utilized by *de novo* glycerolipid biosynthesis (e.g., Kennedy pathway; Weiss et al., 1960) to sequentially acylate glycerol-3-phosphate producing first *de novo sn*-1/2-diacylglycerol (DAG) containing two 18:1-OH, and subsequently triacylglycerol (TAG) molecular species containing three 18:1-OH (e.g., 3HFA-TAG; Bafor et al., 1991; Lager et al., 2013; Bates, 2016, 2022). However, *P. fendleri* predominantly accumulates TAG species containing only two lesquerolic acids (2HFA-TAG), with each HFA strictly localized to the *sn*-1 and *sn*-3 positions of TAG (Hayes et al., 1995; Hayes and Kleiman, 1996). In *P. fendleri*, the 18:1-OH produced on PC is also incorporated into the acyl-CoA pool through acyl editing, but it is further elongated to 20:1-OH prior to incorporation into TAG (Reed et al., 1997; Moon et al., 2001). Until recently, the pathway of 20:1-OH incorporation into TAG was previously unclear. A Kennedy pathway similar to castor would make logical sense to limit unusual fatty acids in membrane lipids, which adversely affect membrane structure–function (Millar et al., 2000). However, transcriptomic studies on developing *P. fendleri* seeds indicated high expression of phosphatidylcholine:diacylglycerol cholinephosphotransferase (PDCT; Kim and Chen, 2015; Horn et al., 2016), which is a hallmark of TAG biosynthesis from PC-derived DAG (rather than *de novo* DAG of the Kennedy pathway) in Brassica species (Lu et al., 2009; Bates and Browse, 2011, 2012; Yang et al., 2017; Bai et al., 2020). To synthesize 2HFA-TAG by a PC-derived DAG pathway utilizing PDCT, *de novo* DAG containing *sn*-1 20:1-OH would need to move through the membrane lipid PC pool to produce a PC-derived DAG containing *sn*-1 20:1-OH. Subsequent *sn*-3 acylation with 20:1-OH would generate the 2HFA-TAG molecular species. However, analysis of lipid fluxes in HFA-producing Arabidopsis and characterization of *Camelina sativa* PDCT properties suggested that PDCT enzymes discriminate against HFA-containing substrates for PC synthesis (Bates and Browse, 2011; Lager et al., 2020). In addition, lesquerolic acid had not been found in *P. fendleri* seed PC (Chen et al., 2011), thus making it unclear if a PDCT promoted PC-derived DAG pathway of TAG biosynthesis exists in *P. fendleri.*

Recently we utilized *in vivo* isotopic labeling of lipid metabolism in developing *P. fendleri* embryos to trace the pathway of newly synthesized fatty acids and glycerol backbones through the lipid metabolic network into different TAG molecular species (Bhandari and Bates, 2021). This lipid flux analysis demonstrated a novel TAG biosynthetic pathway in *P. fendleri* that combined PC-derived DAG production with TAG remodeling as the major mechanism to accumulate 2HFA-TAG while limiting HFAs in membrane lipids (Supplementary Figure S1). *Physaria fendleri* TAG biosynthesis elucidated by the isotopic tracing starts with the use of PC-derived DAG that does not contain HFA for the *sn*-3 acylation with 20:1-OH, producing initially a TAG molecular species containing only one HFA (1HFA-TAG). The isotopic tracing further indicated the glycerol backbone and HFA of 1HFA-TAG are converted into 2HFA-TAG over time. The proposed mechanism is that 1HFA-TAG is remodeled into 2HFA-TAG involving a TAG lipase that removes the *sn*-1 non-HFA from 1HFA-TAG transiently producing *sn*-2-acyl-*sn*-3-HFA-DAG. Subsequent acylation with 20:1-OH at the *sn*-1 position generates the final 2HFA-TAG (Supplementary Figure S1; Bhandari and Bates, 2021). Thus, the isotopic tracing revealed how *P. fendleri* can utilize PDCT activity for a PC-derived DAG pathway of TAG biosynthesis, without the need to move lesquerolic acid through the PC membrane lipid pool.

While the *P. fendleri* lipid metabolic tracing indicated the major pathway of carbon flux into TAG, the identity of the enzymes involved are unclear. Especially, the TAG lipase at the center of the TAG remodeling pathway. TAG lipases in plants have multiple roles, including TAG turnover during: oilseed germination to provide energy and carbon for seedling establishment; pollen tube growth to provide fatty acids for membrane lipid production; and senescence or various stresses that produce transient TAG pools from membrane lipid turnover (Theodoulou and Eastmond, 2012; Troncoso-Ponce et al., 2013; Kelly and Feussner, 2016; Pyc et al., 2017; Shimada et al., 2017; Wang et al., 2019; Ischebeck et al., 2020). Developing *P. fendleri* seeds express 12 genes annotated as TAG lipases (Horn et al., 2016), including a homolog of the *SUGAR DEPENDENT 1* (SDP1) TAG lipase. SDP1 was first characterized as the dominant TAG lipase involved in TAG turnover during seed germination in *Arabidopsis thaliana* (Eastmond, 2006; Kelly et al., 2011). Yet, *SDP1* expression increases during seed development in preparation for germination, which leads to a reduction in total seed TAG during seed maturation. The suppression of *SDP1* during seed development has led to increased levels of seed oil in Arabidopsis (Van Erp et al., 2014), *Brassica napus* (Kelly et al., 2013), jatropha (Kim et al., 2014), and soybean (Kanai et al., 2019; Aznar-Moreno et al., 2022).

The proposed TAG remodeling pathway of oil biosynthesis in *P. fendleri* involves a TAG lipase (Bhandari and Bates, 2021); therefore, we considered the possibility that PfeSDP1 may be involved in TAG remodeling during seed development, and/or SDP1 may be involved in TAG turnover during seed germination as previously characterized in other species through three hypotheses (Supplementary Figure S1). In hypothesis 1, if PfeSDP1 was involved in TAG remodeling then there would be two key features of PfeSDP1: (1) the enzymatic selectivity for TAG molecular species would either favor non-HFA, or be non-selective to TAG molecular species so as to not preferentially turnover the major 2HFA-TAG molecular species produced during seed development and (2) that the suppression of *PfeSDP1* during seed development would lead to increased accumulation of 1HFA-TAG at the expense of 2HFA-TAG reducing the total HFA content of the seed oil. In hypothesis 2, if the role of PfeSDP1 is similar to that of SDP1 in other species being primarily for TAG turnover during germination, then the enzymatic activity may favor turnover of 2HFA-TAG, and that suppression of *PfeSDP1* during seed development would increase total seed TAG without affecting fatty acid composition. In hypothesis 3, if PfeSDP1 was involved in both TAG biosynthesis and TAG turnover during seed maturation, then the enzymatic activity would be non-selective to TAG molecular species, and suppression of PfeSDP1 during seed development would both increase the proportion of 1HFA-TAG and total seed oil. Therefore, to further understand the potential roles of PfeSDP1 in *P. fendleri* lipid metabolism in the context of these three hypotheses we pursued both *in vitro* and *in vivo* studies through enzymatic investigations of heterologously expressed *PfeSDP1*, analysis of SDP1 expression at different developmental stages, and produced seed-specific *PfeSDP1*_RNAi knockdown lines of *P. fendleri*.

## Materials and Methods

### RNA Isolation and Quantitative Real-Time PCR Analysis

*Physaria fendleri* seed pods of 24, 30, and 42 (mature seed) days after pollination (DAP) were harvested to collect developing seeds and immediately frozen in liquid nitrogen and stored at −80°C till used for RNA extraction. Three replicates of 10 seeds per time point including 1 day germinated seeds were used for RNA extraction. Total RNA was extracted using a Spectrum plant total RNA kit (Sigma). Total RNA (10 μg) was treated with RNase-Free DNase (Qiagen) and cleaned using a RNeasy® Mini Kit (Qiagen). One microgram of the RNA was used to generate cDNA using an iScript cDNA synthesis kit (Bio-Rad). Quantitative real-time PCR (qRT-PCR) analyses were carried out with CFX96 Real-Time System (Bio-rad), using Maxima SYBR Green qPCR Master Mix (Thermo Fisher Scientific Co.), and relative expression values were calculated using the ∆-Ct-method. A complete list of the primers used for RT-PCR is presented in Supplementary Table S1.

### Over Expression and Purification of PfeSDP1 Protein

The full-length coding sequence of *PfeSDP1* was ordered from Integrated DNA Technologies (IDT) based on prior developing *P. fendleri* RNAseq results (Horn et al., 2016) and amplified using the gene-specific primers (Supplementary Table S1), PfeSDP1-FW (added *Kpn*I) and PfeSDP1-RV (added *Not*I). The amplified product was excised with *Kpn*I and *Not*I and cloned into the yeast expression vector, pYES2/NT-C (Invitrogen), under the control of galactose-inducible promoter, GAL1 and with a His-Tag at the N-terminus. The recombinant plasmid, pYES2/NT-C + *PfeSDP1* and empty vector (pYES2/NT-C) was transformed into *Saccharomyces cerevisiae* expression strain, INVScI by using lithium acetate method as described in the Invitrogen user manual. The yeast transformants were grown overnight in yeast synthetic media without uracil (SM-URA) + 2% glucose, and the transgene was induced by adding 0.4 A_600_ overnight culture to 500 ml of induction media (SM-URA + 2% galactose +1% raffinose). After 36 h of shaking at 220 rpm at 28°C, the cells were harvested by centrifugation and resuspended in the lysis buffer [50 mM sodium phosphate buffer pH 8.0; 250 mM NaCl; 5% glycerol; 2 mM sodium taurodeoxycholate, 1 mM phenylmethylsulfonyl fluoride (PMSF) and 1x ProteaseArrest™ for yeast/fungal; G-biosciences, United States]. The cells were lysed with 0.5 mm Zirconia/Silica beads using a bead beater (BioSpec Products, United States), and the resulting cell lysate was subjected to centrifugation at 20,000 rpm for 20 min to remove cell debris and glass beads. Recombinant SDP1 protein was purified under native conditions using ProBond™ purification system (Invitrogen, United States). Briefly, the supernatant from cell lysate was loaded on to Ni-NTA column and the protein was eluted using 250 mM imidazole according to the manufacturer’s instructions. Two millimolar sodium taurodeoxycholate was included in all the buffers. The eluted protein fractions were pooled after SDS-PAGE (10%) analysis and subjected to concentration and buffer exchange (50 mM sodium phosphate pH 8.0, 2 mM sodium taurodeoxycholate, and 10% glycerol) using Amicon Ultra-15 centrifugal unit (Millipore Sigma, United States; Cat. no: UFC903008). Recombinant purified protein was confirmed by immunoblotting using anti 6X-His Tag monoclonal antibody (HIS.H8; Invitrogen, United States) produced in mouse at a dilution of 1:3000(v/v) and rabbit anti-mouse IgG secondary antibody tagged with HRP (Invitrogen, United States) at a dilution of 1:5,000 (v/v). Blots were developed with Clarity™ western ECL substrate (BIO-RAD, United States). Protein content was determined using Pierce BCA protein assay kit (Thermo Scientific, United States) using BSA as standard.

### *In vivo* Metabolic Labeling and Neutral Lipid Profiling of Yeast Expressing PfeSDP1

Yeast transformants harboring pYES2/NT-C + *PfeSDP1* and empty vector (pYES2/NT-C) were pre-cultured overnight in SM-URA + 2% glucose. For *in vivo* labeling, cells at an A_600_ of 0.4 were inoculated into an induction medium (SM-URA + 2% galactose +1% raffinose) containing 0.2 μCi/ml [^14^C]acetate (American Radiolabeled Chemicals, Inc., United States) and grown at 28°C up to stationary phase. Total Lipid extraction with chloroform:methanol:2% phosphoric acid (2:1:1, v/v/v) was performed on yeast cells, pellet from a volume of culture in ml x A_**600**_ = 40. Neutral lipids were separated on sillica TLC plates (Analtech Silica gel HL; 20 cm × 20 cm; 250 μm thickness; 15 μm particle size) using petroleum ether:diethyl ether:acetic acid (70:30:1, v/v/v) as a solvent system. The labeled neutral lipids were measured on a Typhoon FLA 7000 phosphor imager and the relative amount of radioactivity in TAG, free fatty acid (FFA), and DAG were using quantified ImageQuant TL 7.0 (GE healthcare, United States).

### Preparation of 1HFA- and 2HFA-TAG Substrates

[^14^C]1HFA- and [^14^C]2HFA-TAG substrates for *in vitro* lipase assay were purified using thin layer chromatrography (TLC) from total lipids extracts of *P. fendleri* developing embryos which are cultured in the presence of [^14^C]acetate as described in Bhandari and Bates (2021). Unlabeled [^12^C]2HFA-TAG was purified using TLC from Physaria seed oil as per previously defined (Bhandari and Bates, 2021). The purified substrates are quantified by GC-FID as described below.

### *In vitro* Lipase Activity Assays

Lipase activity of PfeSDP1 was measured by monitoring the release of [^14^C]fatty acid from [^14^C]TAG. The assay was carried out in a reaction volume of 250 μl consisting of 50 mM Tris-(HCl) pH 8.0, 2 mM DTT, 2 mM sodium taurodeoxycholate, and 2 mM CaCl_2_. A total of 0.02 μCi of [^14^C]triolein (0.363 nmol; American Radiolabeled Chemicals, Inc., United States) or [^14^C]1HFA-TAG (1 nmol) or [^14^C]2HFA-TAG (1 nmol) dissolved in ethanol was added to the reaction buffer and sonicated using water bath sonicator to disperse the lipid. For competitive lipase assay, equimolar ratio (1:1) of [^14^C]triolein:[^12^C]triolein or [^14^C]triolein:[^12^C]2HFA-TAG was dissolved in ethanol and added to reaction buffer. The reaction was started by the addition of 50 μg recombinant protein and incubated at 30°C for 45 min with shaking (1,000 rpm). The assay was stopped by adding chloroform:methanol:acetic acid (2:1:0.1, v/v/v). After vortex and centrifugation, chloroform phase was transferred into a new tube and dried using nitrogen evaporator. Dried lipids were solubilized in 40 μl chloroform and separated and neutral lipids separated by TLC and quantified by phosphor imaging as above.

### RNAi Constructs Preparation

*PfeSDP1* RNAi target gene fragments of 240 bp were PCR amplified using synthesized SDP1 DNA (IDT) as a template with Phusion Taq (New England Biolabs). The primers used for the PCR reaction were: PfeSDP1-RNAiF (added *Sac*II and *Not*I) and PfeSDP1-RNAiR (added *Pst*I and *Xma*I restriction sites). The forward arm was cloned at *Not*I/*Xma*I, whereas the reverse arm was cloned at *Pst*I/*Sac*II sites of the J2 vector under seed-specific 2S albumin promoter (Shockey et al., 2015). This clone was sequence-verified, and the promoter:gene:terminator cassette was released by digestion with *Asc*I and cloned into binary vector pB9 (Shockey et al., 2015). A complete list of the primers used for RT-PCR is presented in Supplementary Table S1.

### Tissue Culture and Transformation

Plant transformation was performed using the Agrobacterium GV3101 strain carrying the *P. fendleri* SDP1-RNAi construct in binary vector pB9 (Shockey et al., 2015). The *in vitro P. fendleri* grown plant’s mature leaves were used for the transformation. Tissue culture and transformation protocol followed as described previously (Chen, 2011) with some modifications, using Basta 1.2 mg/L for transgenic selection. The leaves were harvested from the *in vitro* grown plants in sterile conditions and cut into four pieces. The agrobacterium solution in half strength Murashige and Skoog (MS) harbored SDP1-RNAi construct for 5 min with gentle shaking. Following inoculation, the excess Agrobacterium solution was removed by soaking on sterilized filter paper. Leaf segments were transferred to the callus and shoot induction media (CSI) composed of basal media (BM; 1/2 strength MS with 3% sucrose, 0.6% agar, pH 5.7), supplemented with 0.1% 6-BA (6-benzylaminopurine) and 0.01% NAA (naphthaleneacetic acid). Co-cultivated in the dark for 2 days (to increase transformation efficiency) and then 2 days in the light. After 2 days of incubation under light, leaves were again cut into 5 mm sizes and transferred on CSI media containing 0.6 mg/L Basta for transgenic selection and 100 mg/L timentin to repress agrobacterium growth. After 6–8 weeks, Basta resistant yellowish-green calli come from the leaf segments were cut into small pieces (each calli from the leaf segment were marked as an independent line) and transferred on CSI media containing 1.2 mg/L Basta for strong selection. After four rounds of successive selection to get rid of chimeric tissues, shoots were subculture on BM supplemented with 1 mg/L 6-BA, 1 mg/L indole-3-butyric acid (IBA), and 1.2 mg/L Basta for the shoot induction. After 3–4 weeks, shoots of 10–15 mm were transferred to BM supplemented with 1 mg/L IBA and 1.2 mg/L Basta for rooting. After 3–5 weeks, shoots with 3–5 roots were carefully removed from the media and dipped into an antifungal solution of Terbinafine hydrochloride (1 μM) and then transferred to the soil pot (2” × 2”). After root establishment, the plants were transferred to larger pots (4” × 4”) in growth room set at 16/8 h light/dark cycle with a light intensity of approximately 250 μmol m^−2^ s^−1^, 23°C–24°C temperature, and 50% humidity. Seeds from T1 selfed plants (T2 seed) were obtained after forced pollination using the pollen of different flowers from the same plant.

### Germination Assay

Around 20–25 seeds for each genotype were surface sterilized with ethanol for 1 min, washed with sterilized water three times, transferred to wet blotting papers in half-strength MS, and kept in the dark for 2 days to germinate. After germination, the plates were transferred under the light. The seedling establishment percentage was calculated after 4 days in light (16 h light/8 h dark), defined by the development of the green cotyledonary leaves.

### Quantification of Different TAG Species by HPLC and GC-FID

Total lipids were extracted from ~30–40 mg mature seeds using hexane-isopropanol method (Hara and Radin, 1978) with some modification as described in Shockey et al. (2019). Extracted lipids were dried using N_2_ gas and dissolved in hexane:isopropanol (60:40) for separation of different TAG species (0, 1, and 2HFA-TAGs) by high performance liquid chromatography (HPLC) on an Agilent 1260 Infinity II LC with YMC Pack PVA-SIL (250 mm × 4.6 mm, 5 μm particle size) column. The HPLC instrumentation setup, gradient, and fraction collection methods for different TAG species are reported previously (Kotapati and Bates, 2020). The collected fractions were dried under nitrogen gas after adding 25 μg of internal standard, Tri-17:0 TAG (Nucheck Prep, United States) and converted to fatty acid methyl esters (FAMEs) using 2.5% H_2_SO_4_ in methanol at 85°C for 1 h. FAMES were extracted into hexane after phase separation with 0.88% potassium chloride and quantified with Agilent 7,890 Gas Chromatograph with Flame Ionization Detection (GC-FID) on a DB-HeavyWAX column (30 m, 0.25 mm internal diameter, and 0.25 μm film thickness). The GC-FID conditions are as follows: split mode injection (1:20), 5 μl injection volume, injector at 250°C and FID at 270°C, with oven temperature programmed at 140°C for 0 min, ramped at the rate of 20°C per min to 200°C, then increasing at 6°C per min to 270°C and holding at 270°C for 5 min. Three replicates of five seeds for each SDP1_RNAi line and control were used for lipid extraction and analysis.

## Results

### *Physaria fendleri* SDP1 Is Evolutionary Conserved and Highly Expressed During Late Seed Development and Germination

**Figure 1:**
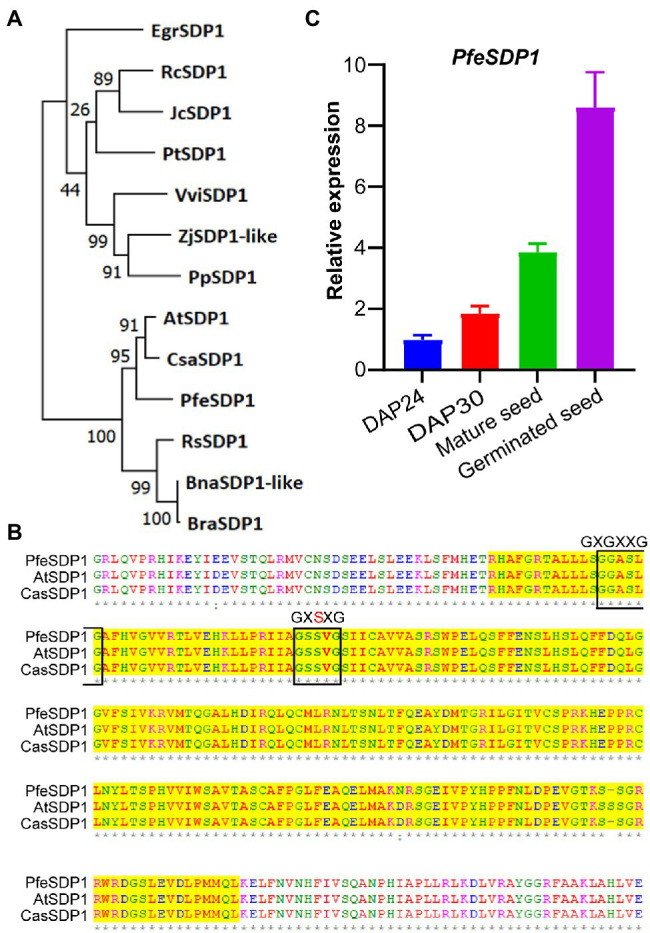
Homology of *Physaria fendleri* SDP1 and expression in seed tissue. **(A)** Phylogenetic analysis of *P. fendleri* and other SDP1 proteins. The phylogenetic tree was constructed using MEGA11. Numbers at the tree branches indicate percent bootstrap support of 1,000 iterations. Species abbreviations and accession numbers of each protein given in Supplementary Table S2. **(B)** An amino acid alignment of patatin domain of *P. fendleri* with *Arabidopsis* and *Camelina* SDP1. Lipase motifs GXGXXG and GXSXG are highlighted with a black box. Protein aliment was performed using ClustalW. **(C)** Relative expression of the *PfeSDP1* gene during seed development and germinated seeds, the expression quantified by qRT-PCR and normalized to the reference *18S* gene. DAP24; DPA30; indicates 24 and 30 days after pollination, respectively. Expression values are the average of three biological replicates ± SE.

A full-length putative *SDP1* gene sequence from *P. fendleri* was identified from the developing seed transcriptome based on homology to *AtSDP1* (Horn et al., 2016). Phylogenetic analysis revealed that *P. fendleri* SDP1 is highly conserved among the Brassicaceae family, showing 89% and 90% amino acid sequence identity with *Camelina* and *Arabidopsis* SDP1 homologs, respectively (Figure 1A; Supplementary Table S2). Protein sequence alignment contains a conserved patatin-like domain with its close relative Arabidopsis and Camelina containing lipase motifs GXGXXG and GXSXG (Figure 1B). Arabidopsis *SDP1* is expressed during late seed development and germination (Eastmond, 2006), and prior transcriptomics of *P. fendleri* only reported on developing seeds, of which *SDP1* was expressed but low relative to other putative TAG lipases (Horn et al., 2016). Therefore, we quantified the relative *SDP1* expression during *P. fendleri* seed development and germination by qRT-PCR. *SDP1* expression increased from 24 to 30 DAP and was almost four-fold higher in mature seeds than at 24 DAP (Figure 1C). The *SDP1* expression further increased 8-fold compared to 24 DAP 1 day after seed germination (Figure 1C).

### Characterization of Triacylglycerol Lipase Activity of PfeSDP1

**Figure 2:**
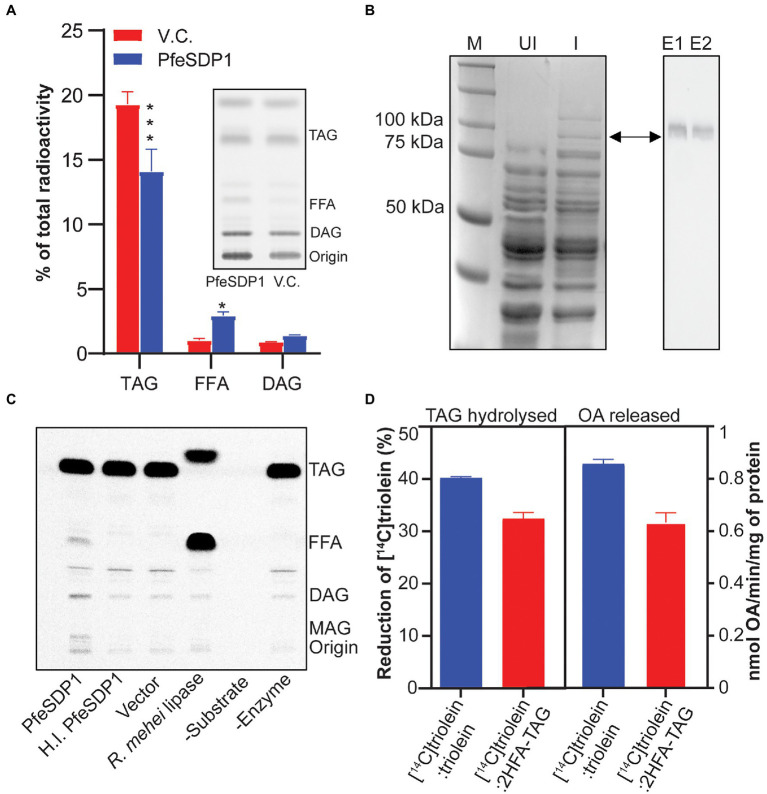
Characterization of PfeSDP1 triacylglycerol lipase activity. **(A)** PfeSDP1 overexpressed in *S. cerevisiae* and *in vivo* metabolic labeling with [^14^C]acetate to label fatty acid synthesis. The relative amount of radioactivity in TAG, FFA, and DAG neutral lipids quantified by thin-layer chromatography (TLC) separation and phosphor imaging (inset). **(B)** Purification of a His-tagged PfeSDP1 (96 KDa) expressed in *S. cerevisiae*. Left, SDS-PAGE of total protein from uninduced (UI) cells and induced (I) cells. M, protein marker. Right, Western blot with anti-(His)6 antibody from E1 and E2, elution fractions of purified protein from Ni-NTA column. **(C)** TAG lipase activity of purified PfeSDP1 protein. Phosphor image TLC of *in vitro* lipase assay reactions with [^14^C]triolein as substrate. Lipase from *Rhizopus mehei* was used as positive control. Elution fraction from vector control (VC), heat inactivated (HI.) PfeSDP1 and no enzyme addition was used as negative controls. **(D)** Competitive lipase assay of PfeSDP1 protein with equimolar amounts (1:1) of [^14^C]triolein:[^12^C]triolein, or [^14^C]triolein:[^12^C]2HFA-TAG as substrates. Lipase activity was represented as amount (%) of [^14^C]triolein degraded (left) and release of [^14^C]oleic acid (right) as measured by phosphor imaging. TAG, triacylglycerol; FFA, free fatty acids; DAG, diacylglycerol; ST, sterol; MAG, monoacylglycerol; PL, phospholipids; OA, oleic acid. All the values are represented as Mean ± SD of three independent experiments and significant difference between wild-type control and PfeSDP1 knock-down lines was determined using two-way ANOVA (***p ≤ 0.001 and *p ≤ 0.33).

To confirm the TAG lipase activity and evaluate possible fatty acid selectivity of the putative SDP1 gene from *P. fendleri*, we overexpressed PfeSDP1 protein in yeast strain, INVSc1 under GAL1 induction promoter and examined its lipolytic activities in *in vivo* by metabolic labeling using [^14^C]acetate. Analysis of neutral lipids from whole-cell total extracts indicated the level of TAG decreased significantly in the cells expressing PfeSDP1 compared to the cells harboring empty vector. Concurrently, the levels of DAG and FFA were increased in PfeSDP1 overexpressed cells compared to vector control (Figure 2A), consistent with TAG lipolytic activity of PfeSDP1 in *in vivo*. We also tested the *in vitro* TAG lipase activity of PfeSDP1 by using a His-tagged recombinant protein overexpressed in INVSc1 and purified on Nickel-NTA agarose beads (Figure 2B). SDS-PAGE analysis of the purified fusion protein indicated a protein band of expected size (~96 kDa), which was further confirmed by western blotting (Figure 2B, right panel). The His-tagged fusion protein was directly used for enzymatic characterization using [^14^C]triolein as a substrate. Isolated PfeSDP1 could hydrolyze [^14^C]triolein to FFA and DAG similar to *Rhizopus mehei* lipase (positive control; Figure 2C). This validated the reduction of TAG and accumulation of FFA, and DAG phenotype observed in the *in vivo* metabolic labeling study. No activity was detected in the vector and heat-inactivated protein controls. As *P. fendleri* seed oil is composed of more HFA-containing TAG molecular species compared to TAG only containing common fatty acids, we also tested its activity against HFA-containing TAG substrates and found that PfeSDP1 can hydrolyze [^14^C]TAG with both 1 and 2 HFA (Supplementary Figure S2). To further characterize the common vs. HFA-TAG substrate preference of PfeSDP1, we carried out a competitive *in vitro* lipase assay using the equimolar ratio (1:1) of [^14^C]triolein:[^12^C]triolein or [^14^C]triolein:[^12^C]2HFA-TAG. The substrate specificity was determined by measuring the rate of [^14^C]triolein degradation and the amount of [^14^C]oleic acid released. In the assay, if PfeSDP1 has a higher selectivity for 2HFA-TAG the rate of [^14^C]triolein degradation should decrease relative to the [^14^C]triolein:[^12^C]triolein control. However, if PfeSDP1 prefers triolein over 2HFA-TAG, we would expect the rate of [^14^C]triolein degradation to increase. When the PfeSDP1 activities in the presence of equimolar mixtures of substrates were compared, the rate of [^14^C]triolein degradation and the amount of oleic acid released was decreased by ~20% and ~26%, respectively, in the presence of HFA-containing TAG compared to common fatty acid-containing TAG (Figure 2D). Together, these results confirm that PfeSDP1 is a TAG lipase and can utilize TAG molecular species containing both common fatty acids and HFAs, but with a preference for HFA-TAG containing molecular species.

### *SDP1* Knockdown Increased Seed Weight With Limited Effect on Post-germination Seedling Establishment

**Figure 3:**
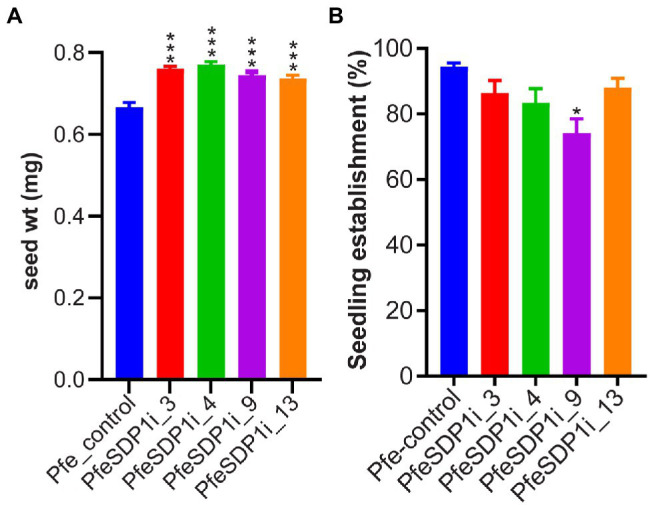
Seed-specific *PfeSDP1_RNAi* increases seed weight and has a limited effect on seed establishment. **(A)** The seed weight of PfeSDP1_RNAi T1 generation plant seeds (T2 seeds) represent the average seed weight (*n* = 10). **(B)** Percentage of seedling establishment after 4 days in light (16 h light/8 h dark), defined by the development of the green cotyledonary leaves. Asterisks (*) and (***) indicate significant differences at *p* ≤ 0.05 and *p* ≤ 0.0005 to the control by two-tailed paired *t*-tests.

To investigate the *in-planta* function of PfeSDP1, we designed an RNAi knockdown construct expressed under the strong seed-specific promoter 2S albumin (Guerche et al., 1990; Shockey et al., 2015) to repress the *SDP1* expression only in developing *P. fendleri* seeds to avoid any possible pleiotropic effects at other developmental stages. The PfeSDP1_RNAi transgenic plants were produced by Agrobacterium transformation of leaf tissue and plant regeneration through tissue culture. Out of 12 regenerated plants, nine transgenic PfeSDP1_RNAi plants were confirmed by PCR using vector and gene-specific primers (Supplementary Figure S3A). Seeds harvested from T1 generation plants (T2 seeds) were utilized to determine the effect of *PfeSDP1* knockdown on seed weight, lipid content, germination, and seedling establishment. Based on increased seed weight the top four transgenic lines were selected (PfeSDPi_3, 4, 9, and 13; Supplementary Figure S3B), and the knockdown of *PfeSDP1* expression in these lines was confirmed by qRT-PCR of RNA extracted from the germinating T2 seeds (Supplementary Figure S4). These four selected transgenic lines were used for further analysis in this study. The PfeSDP1_RNAi knockdown lines had a significant increase in seed weight ranging from 0.74–0.77 mg (12%–16%) in line 13 and line 3, respectively, compared to the wild-type average seed weight of 0.66 mg (Figure 3A). No considerable difference in seed germination was measured, and there was a limited effect on post-germination seedling establishment in the PfeSDP1_RNAi plants compared to control plants (94% seedling establishment), except for line 9 which had 74% seedling establishment (Figure 3B).

### *SDP1* Suppression Increased Both Total Oil Content and Hydroxy Fatty Acid Content

**Figure 4:**
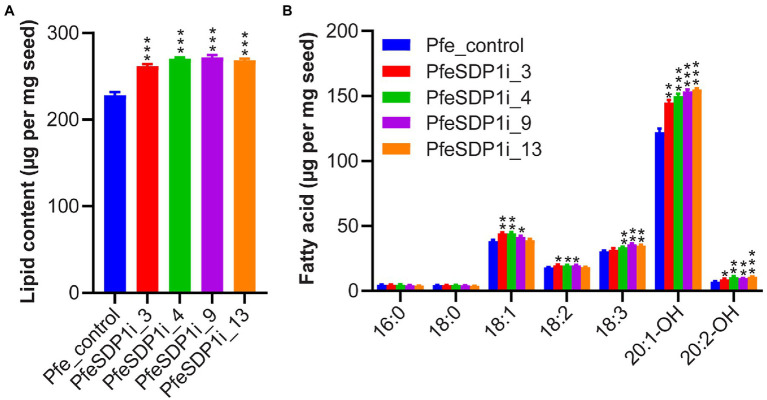
Total fatty acid amount increased in *Physaria fendleri* SDP1_RNAi seeds. **(A)** Total fatty acid content of PfeSDP1_RNAi lines, data represents mean ± SEM (*n* = 3–10) per line. **(B)** Individual fatty acid content of lines in **(A)**. Additional minor fatty acids (18:1-OH, 18:2-OH, 20:0, 20:1) each representing less than 1% of total fatty acids are included in Supplementary Table S2. Asterisks (*), (**), and (***) indicate significant differences at *p* < 0.05, *p* ≤ 0.002, and *p* ≤ 0.0006 compared to their respective control by two-tailed paired *t*-tests.

**Figure 5:**
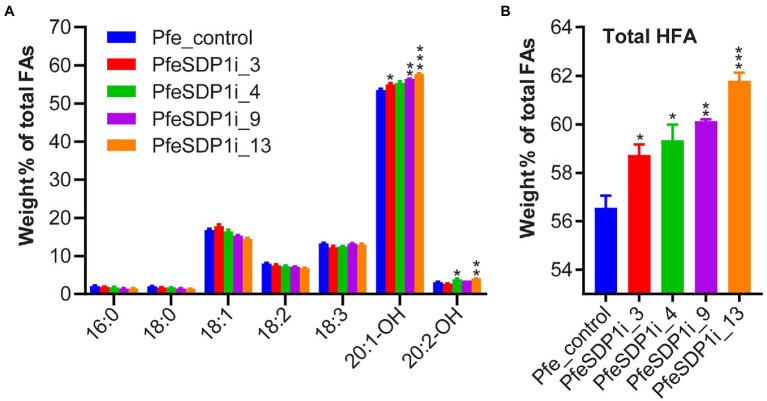
Increased proportion of hydroxy fatty acids in PfeSDP1_RNAi seeds. **(A)** Weight percent of total fatty acids (FA) of PfeSDP1_RNAi lines 3, 4, 9, and 13. **(B)** Total HFAs combined (18:1-OH, 18:2-OH, 20:1-OH, and 20:2-OH). Data represents mean ± SEM (*n* = 3–10) per line. Additional minor fatty acids (18:1-OH, 18:2-OH, 20:0, 20:1) each representing less than 1% of total fatty acids are included in Supplementary Table S3. Asterisks (*), (**), and (***) indicate significant differences at *p* ≤ 0.05, ≤0.002, and ≤ 0.0006 compared to their respective control by two-tailed paired *t*-tests.

The total seed fatty acid content and fatty acid composition was quantified by gas chromatography in seeds (T2) produced from the top selected T1 SDP1_RNAi lines. All four of the top SDP1_RNAi lines had a significant increase in total seed fatty acid content, with total fatty acid content increasing 14%–19% to 261–271 μg per mg seed in line 3 and line 9, respectively, compared to the control seeds containing an average of 228 μg per mg seed (Figure 4A; Supplementary Table S3). The increase in total fatty acid content was attributed primarily to the HFAs 20:1-OH and 20:2-OH, which increased significantly in the transgenic lines to 144.8–155 and 8.9–10.8 μg per mg seed, whereas control seeds contained only 122.2 and 7 μg per mg seed, respectively (Figure 4B; Supplementary Table S4). In addition, *SDP1* knockdown lines had a slight but significant increase in the 18 carbon fatty acids 18:1, 18:2, and 18:3, which increased to 41.6–44.3, 18.2–19.8, and 31.6–35.9 μg per mg seed, whereas control seeds had 38.3, 18.1, and 30.4 μg per mg seed, respectively (Figure 4B; Supplementary Table S4). When the seed fatty acid composition is considered as a weight percentage of total fatty acids, there was a significant increase in total HFA (4%–6%). In contrast, there was no substantial proportional change in individual non-HFAs compared to the control (Figures 5A,B).

**Figure 6:**
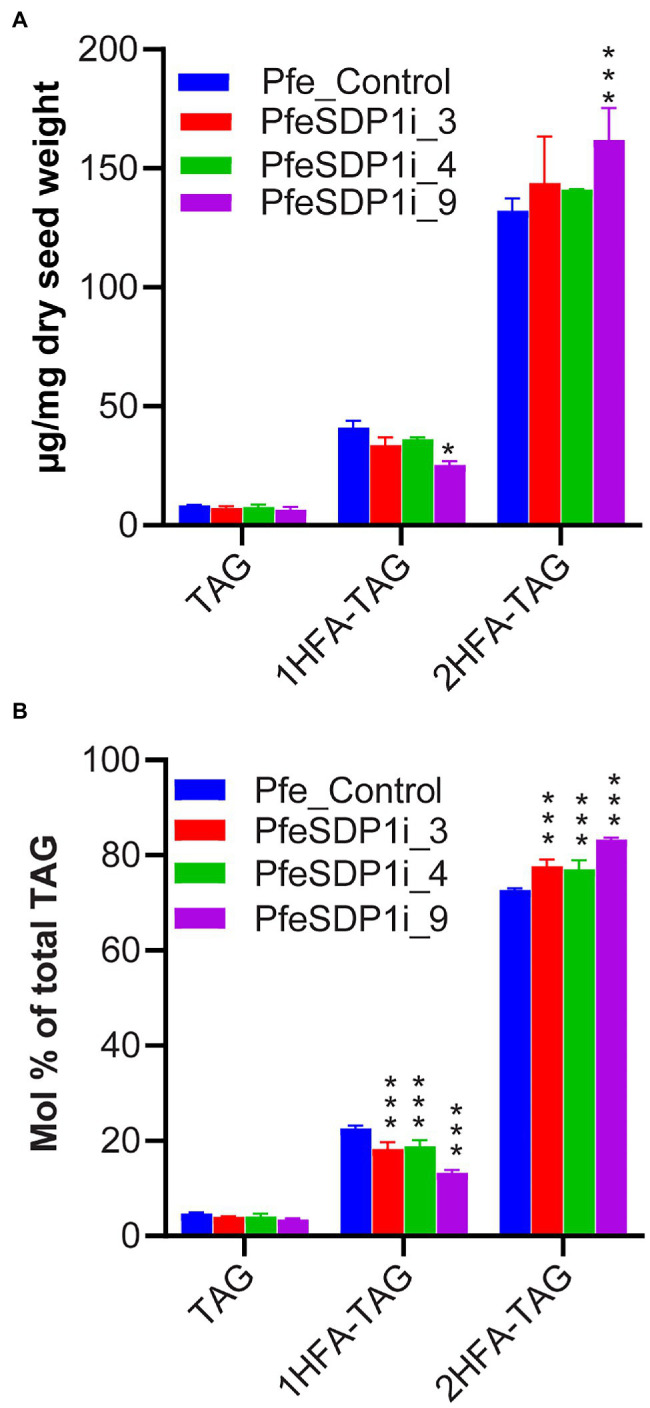
Analysis of different TAG species in the seeds of SDP1 RNAi lines. The levels of different TAG molecular species were expressed as μg/mg dry seed weight **(A)** and relative percent (mol%) of total TAG **(B)**. 1HFA-TAG and 2HFA-TAG represents TAG with one and two hydroxy fatty acids, respectively. Significant difference between wild-type control and PfeSDP1 knock-down lines was determined using two-way ANOVA (***p ≤ 0.001 and *p ≤ 0.33).

Previous research has indicated that *P. fendleri* accumulates TAG molecular species groups of 0HFA-, 1HFA-, or 2HFA-TAG, and that 1HFA-TAG is a metabolic precursor to 2HFA-TAG through TAG remodeling (Supplementary Figure S1). Therefore, to determine if the changes in seed fatty acid content and composition are due to differential accumulation of various TAG molecular species, total lipids were extracted from three independent plants of SDP1 RNAi lines, PfeSDP1i_3, PfeSDP1i_4, and PfeSDP1i_9 and the non-hydroxy-, 1HFA-, and 2HFA-TAG molecular species were separated and collected by HPLC and quantified by GC. No significant changes in the levels of non-hydroxy-TAG were observed between control plants and PfeSDP1 RNAi plants. The amount of 1HFA-TAG in all the PfeSDP1 RNAi lines was significantly reduced compared to the control by 1.2–1.6-fold (Figure 6A), representing a ~12.7% to ~40.7% decrease in the relative proportion of total TAG species (Figure 6B). The amount of 2HFA-TAG was significantly increased by 1–1.2-fold compared to control plants, representing an ~4.7% to ~14.8% increase in the relative proportion of total TAG species (Figure 6). Other than the amount of TAG molecular species, there were small changes in TAG molecular species fatty acid compositions (Supplementary Figure S5). The largest changes were in 1HFA-TAG, where the levels of 18:1 decreased significantly by ~7.5 to 14.1% in all the SDP1 RNAi lines compared to wild-type and were compensated by mostly increases in 18:0 (Supplementary Figure S5). Overall, reduced seed expression of *PfeSDP1* increased total seed fatty acids and the proportion of HFAs in the seed oil predominantly by the enhanced accumulation of 2HFA-TAG.

## Discussion

**Figure 7:**
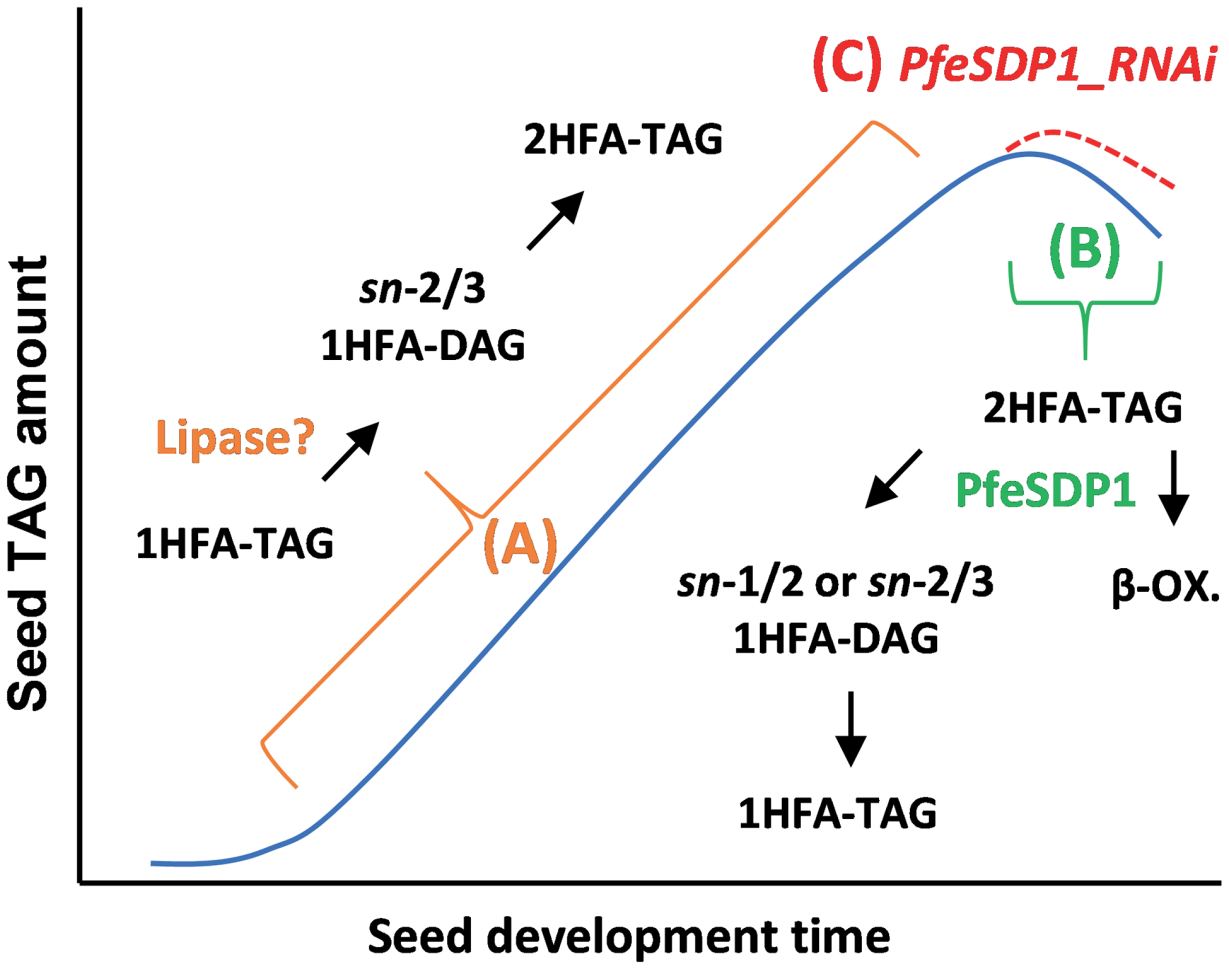
Model of lipase action in *P. fendleri* oil accumulation. In many oilseeds TAG accumulates over seed development, then TAG levels drop ~10%–20% during seed maturation (blue line). **(A)** In *P. fendleri* recent results suggest an uncharacterized lipase is involved in remodeling 1HFA-TAG to 2HFA-TAG during TAG biosynthesis. **(B)** In wild-type *P. fendleri* PfeSDP1 action during late seed development and/or seed maturation leads to TAG turnover and the β-oxidation (β-ox.) of fatty acids reducing total oil amount. Some of the products of 2HFA-TAG turnover are remodeled into 1HFA-TAG. **(C)** The *PfeSDP1_RNAi* lines reduce TAG turnover and remodeling of 2HFA-TAG to 1HFA-TAG, thus increasing total oil amount (red dashed line) and HFA amount. Because *PfeSDP1_RNAi* lines only decreased expression of *PfeSDP1* up to 50%, this result suggests a knockout mutation of *PfeSDP1* may further increase seed oil.

*Physaria fendleri* is a burgeoning oilseed crop that has gained considerable interest in basic oilseed research because of the natural presence of the industrially important HFA, lesquerolic acid in its seeds. Our previous attempt to understand the channeling of newly synthesized common and HFA to seed storage lipids using *in vivo* isotopic labeling has demonstrated a novel TAG remodeling pathway in *P. fendleri* with a proposed TAG lipase as a central player (Bhandari and Bates, 2021). Various TAG lipases were previously reported to be expressed in *P. fendleri* during seed development (Horn et al., 2016). Among these, SDP1 is known to be involved in mobilizing TAG during maturation and desiccation stages of seed development, besides its major TAG turnover role during seed germination (Kelly et al., 2013; Kim et al., 2014; Kanai et al., 2019). Therefore, we hypothesized three possible roles of *P. fendleri* SDP1 during seed development. Firstly, if PfeSDP1 is involved in the proposed pathway of TAG remodeling to produce 2HFA-TAG, its knockdown might block 1HFA-TAG remodeling, thereby reducing the accumulation of 2HFA-TAG in its seeds. Secondly, if the role is similar to other oilseeds for TAG mobilization during the desiccation stage of seed development, its suppression might decrease TAG catabolism, specifically 2HFA-TAG and increase total oil and 2HFA-TAG content. Third, PfeSDP1 may be involved in both TAG remodeling and TAG turnover of which the suppression should lead to increased oil with more 1HFA-TAG, and less 2HFA-TAG. To test the above hypotheses, we isolated the *P. fendleri* SDP1 homolog, demonstrated its lipolytic activity and fatty acid substrate selectivity by heterologous expression in yeast and generated seed-specific SDP1_RNAi knockdown lines of *P. fendleri* to understand the effect of its suppression on total oil content and fatty acid composition. Together our results are most consistent with hypothesis 2 (Figure 7).

### PfeSDP1 Is a HFA Selective Lipase Involved in 2HFA-TAG Turnover

Overexpression of PfeSDP1 in yeast confirmed the lipolytic activity of the protein to hydrolyze triacylglycerols. A similar activity specifically for TAG substrates has been reported for SDP1 proteins from Arabidopsis and soybean (Eastmond, 2006; Kanai et al., 2019). Previously soybean SDP1 preferentially cleaved triolein than trilinolein (Kanai et al., 2019), indicating that substrate selectivity of SDP1 may have a role in the mobilization of certain TAG molecular species over others. Similarly, it is evident from our *in vitro* competitive lipase assay that PfeSDP1 preferentially uses HFA-containing TAG as a substrate over common fatty acid-containing TAG (triolein). Although, SDP1 suppression studies in Brassica and Jatropha (Kelly et al., 2013; Kim et al., 2014) increased seed oil content but without any major changes to the fatty acid composition. However, we observed both a significant increase in seed oil accumulation and an increase in the HFA content of the oil. The increase in HFA content in the PfeSDP1_RNAi lines came from enhanced accumulation of 2HFA-TAG, which is the major TAG species. Therefore, this clearly demonstrates the importance of both TAG anabolic and catabolic pathways during *P. fendleri* seed development for the control of both oil amount and oil quality. Based on our initial stated hypothesis 2, the increase in total seed oil and specifically the final 2HFA-TAG molecular species (rather than the TAG remodeling intermediate, 1HFA-TAG) support the role of PfeSDP1 for predominantly mobilization of the major TAG molecular species during seed germination and late seed maturation. Therefore, PfeSDP1 is likely not the lipase involved in remodeling 1HFA-TAG to 2HFA-TAG, which when suppressed would have led to increased 1HFA-TAG (Figure 7A). However, we have demonstrated that the inhibition of TAG degradation, specifically 2HFA-TAG turnover, by suppression (or mutation) of *PfeSDP1*, would be an excellent strategy to enhance the total amount of industrially valuable lesquerolic acid in *P. fendleri* seeds (Figure 7C).

### Connection of SDP1 to TAG Remodeling in *Physaria fendleri* Seeds

According to the pathway of *P. fendleri* 2HFA-TAG production through TAG remodeling elucidated by isotopic labeling in developing seeds (Figure 7A; Bhandari and Bates, 2021), 1HFA-TAG is synthesized from PC-derived DAG, and subsequently, the *sn*-1 common fatty acid is removed by a lipase to generate *sn*-2-acyl-*sn*-3-HFA-DAG that is finally acylated at the *sn*-1 position with a HFA to make 2HFA-TAG. As hypothesized, if *PfeSDP1* is part of TAG remodeling, then the knockdown of SDP1 activity will produce less 2HFA-TAG and more 1HFA-TAG. The SDP1_RNAi lines accumulate more 2HFA-TAG, and thus we have concluded that PfeSDP1 is likely not involved in the dominant TAG remodeling pathway that produces 2HFA-TAG (thus rejection of initial hypotheses 1 and 3). However, surprisingly the SDP1_RNAi lines also had a significant decrease in total 1HFA-TAG (Figure 6A). This result suggests that some of the 1HFA-TAG that accumulates in mature seeds is due to the action of PfeSDP1 and may involve reverse TAG remodeling of 2HFA-TAG that generates at least part of the substrates utilized for 1HFA-TAG biosynthesis (Figure 7B). Reverse TAG remodeling of 2HFA-TAG likely involves PfeSDP1 cleavage of 2HFA-TAG producing 1HFA-DAG and free HFA. Subsequent 1HFA-TAG production may occur either by acylation of the 1HFA-DAG with a common fatty acid or utilization of the HFA for acylation of a non-HFA-containing DAG molecule. Considering total oil is reduced by PfeSDP1 action in wild-type, the degradation of the free HFA and acylation of some of the 1HFA-DAG by a common fatty acid may be the more likely mechanism. Additionally, the formation of 1HFA-TAG (rather than 2HFA-TAG resynthesis) may be favored by reduced *de novo* lesquerolic acid synthesis during late seed maturation. Interestingly, the remaining 1HFA-TAG in the *PfeSPD1*_RNAi lines had decreases in oleic acid (18:1) content (Supplementary Figure S5B). Oleic acid is the substrate for hydroxylation, thus this result is consistent with a reverse TAG remodeling mechanism in wild-type where PfeSDP1 produced 1HFA-DAG is acylated with 18:1 (from *de novo* fatty acid synthesis) during seed maturation, rather than 20:1-OH that is abundantly produced during the seed filling stage (Bhandari and Bates, 2021; Figure 7B). To further elucidate the mechanisms of TAG remodeling in *P. fendleri*, future studies should target lipases other than PfeSDP1 by RNAi to identify the lipase involved in the dominant TAG remodeling mechanism that produces 2HFA-TAG. Further clarification of PfeSDP1 induced reverse TAG remodeling of 2HFA-TAG to 1HFA-TAG may be elucidated by isotopic labeling studies during the late seed maturation phase and/or measurements of PfeFAH12 activity during this stage of development, of which reduced PfeFAH12 activity would favor the acylation of 1HFA-DAG with oleic acid to produce 1HFA-TAG, rather than resynthesis of 2HFA-TAG.

### *PfeSDP1* Suppression as a Tool to Enhance Seed Oil and HFA Content

The expression pattern of *PfeSDP1* in *P. fendleri*, the preferred activity of PfeSDP1 for 2HFA-TAG, and the increased oil content of PfeSDP1_RNAi lines are consistent with Arabidopsis, Jatropha, and soybean *SDP1* (Eastmond, 2006; Kelly et al., 2013; Kim et al., 2014; Aznar-Moreno et al., 2022), where SPD1 activity is involved in TAG turnover during germination to provide free fatty acids for ß-oxidation, thus producing carbon skeletons and energy for post-germination seedling establishment (Eastmond and Rawsthorne, 2000; Eastmond, 2006). The *PfeSDP1* suppression lines do not indicate any major defect in seed germination or post-germination seedling establishment. In this study, RNAi lines with only up to 50% decrease in *SDP1* transcript were produced (Supplementary Figure S4). The limited effect on seedling establishment may suggest that this reduced *PfeSDP1* expression level still provides enough lipase activity for TAG turnover at this stage. Additionally, previous reports indicate that SDP1 activity greatly enhances germination but is not essential for germination due to low activity of other lipases (Kelly et al., 2011). Transcriptomics of developing *P. fendleri* seeds indicated 12 genes annotated as TAG lipases were also expressed, thus it is likely additional lipases also contribute to *P. fendleri* seed germination and seedling establishment. The ability of other lipases to allow sufficient germination and seedling establishment will require analysis of a *PfeSDP1* null mutant. Additionally, based on our results with only up to 50% knockdown, a CRISPR based mutation of PfeSDP1 may be a valuable approach to further increase total seed oil and HFA levels (Figure 7C). If significant germination issues arise in a *PfeSDP1* mutant, combining *PfeSDP1* knockout with a PfeSDP1 construct under control of a germination-specific promoter may be an ideal bioengineering approach to improving seed oil amount and HFA content without any germination defect.

## Conclusion

The seed-specific knockdown of the HFA-selective TAG lipase *PfeSDP1* demonstrated a significant increase in seed weight and increased seed oil content, and more importantly, increased amount of industrially valuable HFA. In addition, we demonstrate that PfeSDP1 is not directly involved in the *P. fendleri* TAG remodeling pathway that is utilized to synthesize the dominant TAG molecular species 2HFA-TAG; instead, it may have a minor role in reverse TAG remodeling during seed maturation that produces 1HFA-TAG molecular species from 2HFA-TAG. Thus, the elimination of both the 2HFA-TAG turnover during seed maturation and the minor reverse TAG remodeling role together enhances the total HFA content of *P. fendleri* seeds.

## Data Availability Statement

The original contributions presented in the study are included in the article/Supplementary Material, further inquiries can be directed to the corresponding author.

## Author Contributions

PB conceived the project, procured the funding, agrees to serve as the author responsible for contact, and ensures communication. AA, PP, and PB designed experiments, analyzed the data, and wrote the article. AA and PP performed the experiments. All authors contributed to the article and approved the submitted version.

## Funding

This work is supported by the United States Department of Agriculture National Institute of Food and Agriculture #2020-67013-30899, the Hatch Umbrella Project #1015621, the Multi-State Project #NC1203, and the National Science Foundation #PGRP-IOS-1829365.

## Conflict of Interest

The authors declare that the research was conducted in the absence of any commercial or financial relationships that could be construed as a potential conflict of interest.

## Publisher’s Note

All claims expressed in this article are solely those of the authors and do not necessarily represent those of their affiliated organizations, or those of the publisher, the editors and the reviewers. Any product that may be evaluated in this article, or claim that may be made by its manufacturer, is not guaranteed or endorsed by the publisher.

## References

[ref1] Al-Shehbaz I. A. O'kane S. L. Jr. (2002). Lesquerella is united with Physaria (Brassicaceae). Novon 12, 319–329. doi: 10.2307/3393073

[ref2] Aznar-Moreno J. A. Mukherjee T. Morley S. A. Duressa D. Kambhampati S. Chu K. L.. (2022). Suppression of SDP1 improves soybean seed composition by increasing oil and reducing undigestible oligosaccharides. Front. Plant Sci. 13:863254. doi: 10.3389/fpls.2022.863254, PMID: 35401590 PMC8983916

[ref3] Bafor M. Smith M. A. Jonsson L. Stobart K. Stymne S. (1991). Ricinoleic acid biosynthesis and triacylglycerol assembly in microsomal preparations from developing castor-bean (*Ricinus communis*) endosperm. Biochem. J. 280, 507–514. doi: 10.1042/bj2800507, PMID: 1747126 PMC1130577

[ref4] Bai S. Wallis J. G. Denolf P. Engelen S. Bengtsson J. D. Van Thournout M.. (2020). The biochemistry of headgroup exchange during triacylglycerol synthesis in canola. Plant J. 103, 83–94. doi: 10.1111/tpj.14709, PMID: 31991038 PMC7605783

[ref5] Bates P. D. (2016). Understanding the control of acyl flux through the lipid metabolic network of plant oil biosynthesis. Biochim. Biophys. Acta 1861, 1214–1225. doi: 10.1016/j.bbalip.2016.03.021, PMID: 27003249

[ref6] Bates P. D. (2022). “Chapter 6: The plant lipid metabolic network for assembly of diverse triacylglycerol molecular species,” in Advances in Botanical Research. eds. Rébeillé F. Maréchal E. (Academic Press), 225–252.

[ref7] Bates P. D. Browse J. (2011). The pathway of triacylglycerol synthesis through phosphatidylcholine in Arabidopsis produces a bottleneck for the accumulation of unusual fatty acids in transgenic seeds. Plant J. 68, 387–399. doi: 10.1111/j.1365-313X.2011.04693.x, PMID: 21711402

[ref8] Bates P. D. Browse J. (2012). The significance of different diacylgycerol synthesis pathways on plant oil composition and bioengineering. Front. Plant Sci. 3:147. doi: 10.3389/fpls.2012.00147 22783267 PMC3387579

[ref9] Bhandari S. Bates P. D. (2021). Triacylglycerol remodeling in *Physaria fendleri* indicates oil accumulation is dynamic and not a metabolic endpoint. Plant Physiol. 187, 799–815. doi: 10.1093/plphys/kiab294, PMID: 34608961 PMC8491037

[ref10] Broun P. Boddupalli S. Somerville C. (1998). A bifunctional oleate 12-hydroxylase: desaturase from *Lesquerella fendleri*. Plant J. 13, 201–210. doi: 10.1046/j.1365-313X.1998.00023.x, PMID: 9680976

[ref11] Chen G. Q. (2011). Effective reduction of chimeric tissue in Transgenics for the stable genetic transformation of *Lesquerella fendleri*. HortScience 46, 86–90. doi: 10.21273/HORTSCI.46.1.86

[ref12] Chen G. Q. (2017). Castor and Lesquerella Oils: Production, Composition and Uses. Nova Science Publishers, Inc.

[ref13] Chen G. Q. He X. Mckeon T. A. (2005). A simple and sensitive assay for distinguishing the expression of ricin and *Ricinus communis* agglutinin genes in developing castor seed (*R. communis* L.). J. Agric. Food Chem. 53, 2358–2361. doi: 10.1021/jf040405t, PMID: 15769181

[ref14] Chen G. Q. Lin J.-T. Lu C. (2011). Hydroxy fatty acid synthesis and lipid gene expression during seed development in *Lesquerella fendleri*. Ind. Crop. Prod. 34, 1286–1292. doi: 10.1016/j.indcrop.2010.08.003

[ref15] Cocuron J.-C. Anderson B. Boyd A. Alonso A. P. (2014). Targeted metabolomics of *Physaria fendleri*, an industrial crop producing hydroxy fatty acids. Plant Cell Physiol. 55, 620–633. doi: 10.1093/pcp/pcu011, PMID: 24443498

[ref16] Dierig D. A. Wang G. Mccloskey W. B. Thorp K. R. Isbell T. A. Ray D. T.. (2011). Lesquerella: new crop development and commercialization in the US. Ind. Crop. Prod. 34, 1381–1385. doi: 10.1016/j.indcrop.2010.12.023

[ref17] Eastmond P. J. (2006). SUGAR-DEPENDENT1 encodes a patatin domain triacylglycerol lipase that initiates storage oil breakdown in germinating Arabidopsis seeds. Plant Cell 18, 665–675. doi: 10.1105/tpc.105.040543, PMID: 16473965 PMC1383641

[ref18] Eastmond P. J. Rawsthorne S. (2000). Coordinate changes in carbon partitioning and plastidial metabolism during the development of oilseed rape embryos. Plant Physiol. 122, 767–774. doi: 10.1104/pp.122.3.767, PMID: 10712540 PMC58912

[ref19] Guerche P. Tire C. De Sa F. G. De Clercq A. Van Montagu M. Krebbers E. (1990). Differential expression of the Arabidopsis 2S albumin genes and the effect of increasing gene family size. Plant Cell 2, 469–478. doi: 10.2307/3869096, PMID: 12354963 PMC159903

[ref900] Hara A. Radin N. S. (1978). Lipid extraction of tissues with a low-toxicity solvent. Anal. Biochem. 90, 420–426. doi: 10.1016/0003-2697(78)90046-5 727482

[ref20] Hayes D. G. Kleiman R. (1996). A detailed triglyceride analysis of *Lesquerella fendleri* oil: column chromatographic fractionation followed by supercritical fluid chromatography. J. Am. Oil Chem. Soc. 73, 267–269.

[ref21] Hayes D. Kleiman R. Phillips B. (1995). The triglyceride composition, structure, and presence of estolides in the oils of *Lesquerella* and related species. J. Am. Oil Chem. Soc. 72, 559–569. doi: 10.1007/BF02638857

[ref22] Horn P. J. Liu J. Cocuron J. C. Mcglew K. Thrower N. A. Larson M.. (2016). Identification of multiple lipid genes with modifications in expression and sequence associated with the evolution of hydroxy fatty acid accumulation in *Physaria fendleri*. Plant J. 86, 322–348. doi: 10.1111/tpj.13163, PMID: 26991237

[ref23] Ischebeck T. Krawczyk H. E. Mullen R. T. Dyer J. M. Chapman K. D. (2020). Lipid droplets in plants and algae: distribution, formation, turnover and function. Semin. Cell Dev. Biol. 108, 82–93. doi: 10.1016/j.semcdb.2020.02.014, PMID: 32147380

[ref24] Kanai M. Yamada T. Hayashi M. Mano S. Nishimura M. (2019). Soybean (*Glycine max* L.) triacylglycerol lipase GmSDP1 regulates the quality and quantity of seed oil. Sci. Rep. 9:8924. doi: 10.1038/s41598-019-45331-8, PMID: 31222045 PMC6586785

[ref25] Kelly A. A. Feussner I. (2016). Oil is on the agenda: lipid turnover in higher plants. Biochim. Biophys. Acta 1861, 1253–1268. doi: 10.1016/j.bbalip.2016.04.021, PMID: 27155216

[ref26] Kelly A. A. Quettier A.-L. Shaw E. Eastmond P. J. (2011). Seed storage oil mobilization is important But not essential for germination or seedling establishment in Arabidopsis. Plant Physiol. 157, 866–875. doi: 10.1104/pp.111.181784, PMID: 21825108 PMC3192569

[ref27] Kelly A. A. Shaw E. Powers S. J. Kurup S. Eastmond P. J. (2013). Suppression of the SUGAR-DEPENDENT1 triacylglycerol lipase family during seed development enhances oil yield in oilseed rape (*Brassica napus* L.). Plant Biotechnol. J. 11, 355–361. doi: 10.1111/pbi.12021, PMID: 23171303

[ref28] Kim H. U. Chen G. Q. (2015). Identification of hydroxy fatty acid and triacylglycerol metabolism-related genes in lesquerella through seed transcriptome analysis. BMC Genomics 16:230. doi: 10.1186/s12864-015-1413-8, PMID: 25881190 PMC4381405

[ref29] Kim M. J. Yang S. W. Mao H. Z. Veena S. P. Yin J. L. Chua N. H. (2014). Gene silencing of Sugar-dependent 1 (JcSDP1), encoding a patatin-domain triacylglycerol lipase, enhances seed oil accumulation in *Jatropha curcas*. Biotechnol. Biofuels 7:36. doi: 10.1186/1754-6834-7-36, PMID: 24606605 PMC4016141

[ref30] Kotapati H. K. Bates P. D. (2020). Normal phase HPLC method for combined separation of both polar and neutral lipid classes with application to lipid metabolic flux. J. Chromatogr. B Analyt. Technol. Biomed. Life Sci. 1145:122099. doi: 10.1016/j.jchromb.2020.122099, PMID: 32305707

[ref31] Lager I. Jeppson S. Gippert A.-L. Feussner I. Stymne S. Marmon S. (2020). Acyltransferases regulate oil quality in *Camelina sativa* through both acyl donor and acyl acceptor specificities. Front. Plant Sci. 11:1144. doi: 10.3389/fpls.2020.01144, PMID: 32922411 PMC7456936

[ref32] Lager I. Yilmaz J. L. Zhou X.-R. Jasieniecka K. Kazachkov M. Wang P.. (2013). Plant acyl-CoA:Lysophosphatidylcholine Acyltransferases (LPCATs) have different specificities in their forward and reverse reactions. J. Biol. Chem. 288, 36902–36914. doi: 10.1074/jbc.M113.521815, PMID: 24189065 PMC3873549

[ref33] Lu C. Xin Z. Ren Z. Miquel M. Browse J. (2009). An enzyme regulating triacylglycerol composition is encoded by the ROD1 gene of Arabidopsis. Proc. Natl. Acad. Sci. 106, 18837–18842. doi: 10.1073/pnas.0908848106, PMID: 19833868 PMC2774007

[ref34] Mckeon T. A. (2016). “Chapter 4: Castor (*Ricinus communis* L.),” in Industrial Oil Crops. eds. Mckeon T. A. Hayes D. G. Hildebrand D. F. Weselake R. J. (AOCS Press), 75–112.

[ref35] Millar A. A. Smith M. A. Kunst L. (2000). All fatty acids are not equal: discrimination in plant membrane lipids. Trends Plant Sci. 5, 95–101. doi: 10.1016/S1360-1385(00)01566-1 10707074

[ref36] Moon H. Smith M. A. Kunst L. (2001). A condensing enzyme from the seeds of *Lesquerella fendleri* that specifically elongates hydroxy fatty acids. Plant Physiol. 127, 1635–1643. doi: 10.1104/pp.010544, PMID: 11743108 PMC133568

[ref37] Mutlu H. Meier M. A. R. (2010). Castor oil as a renewable resource for the chemical industry. Eur. J. Lipid Sci. Technol. 112, 10–30. doi: 10.1002/ejlt.200900138

[ref38] Ohlrogge J. Thrower N. Mhaske V. Stymne S. Baxter M. Yang W.. (2018). PlantFAdb: a resource for exploring hundreds of plant fatty acid structures synthesized by thousands of plants and their phylogenetic relationships. Plant J. 96, 1299–1308. doi: 10.1111/tpj.14102, PMID: 30242919

[ref39] Patel V. R. Dumancas G. G. Kasi Viswanath L. C. Maples R. Subong B. J. J. (2016). Castor oil: properties, uses, and optimization of processing parameters in commercial production. Lipid Insights 9, 1–12. doi: 10.4137/LPI.S40233, PMID: 27656091 PMC5015816

[ref40] Pyc M. Cai Y. Greer M. S. Yurchenko O. Chapman K. D. Dyer J. M.. (2017). Turning Over a new leaf in lipid droplet biology. Trends Plant Sci. 22, 596–609. doi: 10.1016/j.tplants.2017.03.012, PMID: 28454678

[ref41] Reed D. W. Taylor D. C. Covello P. S. (1997). Metabolism of hydroxy fatty acids in developing seeds in the genera *Lesquerella* (Brassicaceae) and *Linum* (Linaceae). Plant Physiol. 114, 63–68. doi: 10.1104/pp.114.1.63, PMID: 12223689 PMC158279

[ref42] Shimada T. L. Hayashi M. Hara-Nishimura I. (2017). Membrane dynamics and multiple functions of oil bodies in seeds and leaves. Plant Physiol. 176, 199–207. doi: 10.1104/pp.17.01522 29203559 PMC5761825

[ref901] Shockey J. Lager I. Stymne S. Kotapati H. K. Sheffield J. Mason C.. (2019). Specialized lysophosphatidic acid acyltransferases contribute to unusual fatty acid accumulation in exotic Euphorbiaceae seed oils. Planta 249, 1285–1299. doi: 10.1007/s00425-018-03086-y, PMID: 30610363

[ref43] Shockey J. Mason C. Gilbert M. Cao H. Li X. Cahoon E.. (2015). Development and analysis of a highly flexible multi-gene expression system for metabolic engineering in Arabidopsis seeds and other plant tissues. Plant Mol. Biol. 89, 113–126. doi: 10.1007/s11103-015-0355-5, PMID: 26254605

[ref44] Theodoulou F. L. Eastmond P. J. (2012). Seed storage oil catabolism: a story of give and take. Curr. Opin. Plant Biol. 15, 322–328. doi: 10.1016/j.pbi.2012.03.017, PMID: 22516438

[ref45] Troncoso-Ponce M. A. Cao X. Yang Z. Ohlrogge J. B. (2013). Lipid turnover during senescence. Plant Sci. 205-206, 13–19. doi: 10.1016/j.plantsci.2013.01.004 23498858

[ref47] Vandeloo F. J. Broun P. Turner S. Somerville C. (1995). An oleate 12-hydroxylase from *Ricinus communis* L. is a fatty acyl desaturase homolog. Proc. Natl. Acad. Sci. U. S. A. 92, 6743–6747. doi: 10.1073/pnas.92.15.6743, PMID: 7624314 PMC41405

[ref46] Van Erp H. Kelly A. A. Menard G. Eastmond P. J. (2014). Multigene engineering of triacylglycerol metabolism boosts seed oil content in Arabidopsis. Plant Physiol. 165, 30–36. doi: 10.1104/pp.114.236430, PMID: 24696520 PMC4012589

[ref48] Von Mark V. C. Dierig D. A. (2015). “Germplasm improvement to develop commercially viable lines of the new oilseed crop Lesquerella,” in Industrial Crops (Springer), 315–334.

[ref49] Wang K. Durrett T. P. Benning C. (2019). Functional diversity of glycerolipid acylhydrolases in plant metabolism and physiology. Prog. Lipid Res. 75:100987. doi: 10.1016/j.plipres.2019.100987, PMID: 31078649

[ref50] Weiss S. B. Kennedy E. P. Kiyasu J. Y. (1960). Enzymatic synthesis of triglycerides. J. Biol. Chem. 235, 40–44. doi: 10.1016/S0021-9258(18)69581-X 13843753

[ref51] Yang W. Wang G. Li J. Bates P. D. Wang X. Allen D. K. (2017). Phospholipase Dzeta enhances Diacylglycerol flux into triacylglycerol. Plant Physiol. 174, 110–123. doi: 10.1104/pp.17.00026, PMID: 28325849 PMC5411150

