# Supplementary material for: Suppression of Physaria fendleri SDP1 Increased Seed Oil and Hydroxy Fatty Acid Content While Maintaining Oil Biosynthesis Through Triacylglycerol Remodeling
Source: Front Plant Sci. 2022 Jun 3;13:931310. doi: 10.3389/fpls.2022.931310 (PMC9204166; doi:10.3389/fpls.2022.931310)
Supplement: Supplementary file 1 [file Data_Sheet_1.PDF]

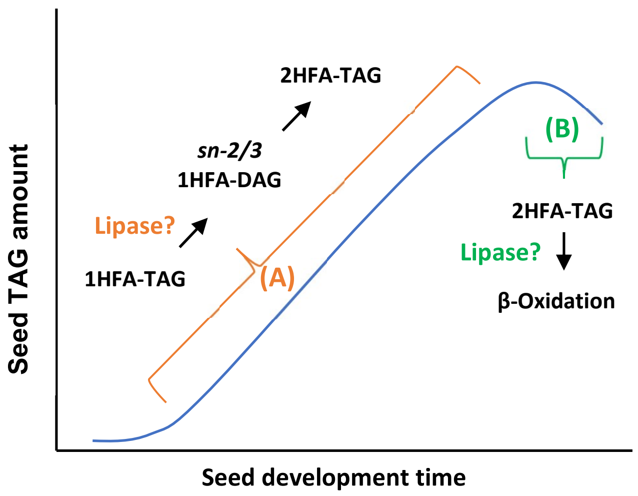

Supplemental Figure S1. Possible roles of lipases in *P. fendleri* oil accumulation.

In many oilseeds TAG accumulates over seed development, then TAG levels drop ~10-20% during seed maturation (blue line). (A) In *P. fendleri* recent results suggest an uncharacterized lipase is involved in remodeling 1HFA-TAG to 2HFA-TAG during TAG biosynthesis (Bhandari and Bates, 2021). (B) The reduction in oil content during seed maturation in several species has been demonstrated to be mostly due to the TAG lipase SDP1. However, considering the dual roles for a lipase in *P. fendleri* TAG biosynthesis and TAG turnover during seed development the possible role of PfeSDP1 in either or both processes is unclear. DAG, Diacylglycerol; HFA, Hydroxy Fatty Acid; TAG, Triacylglycerol.

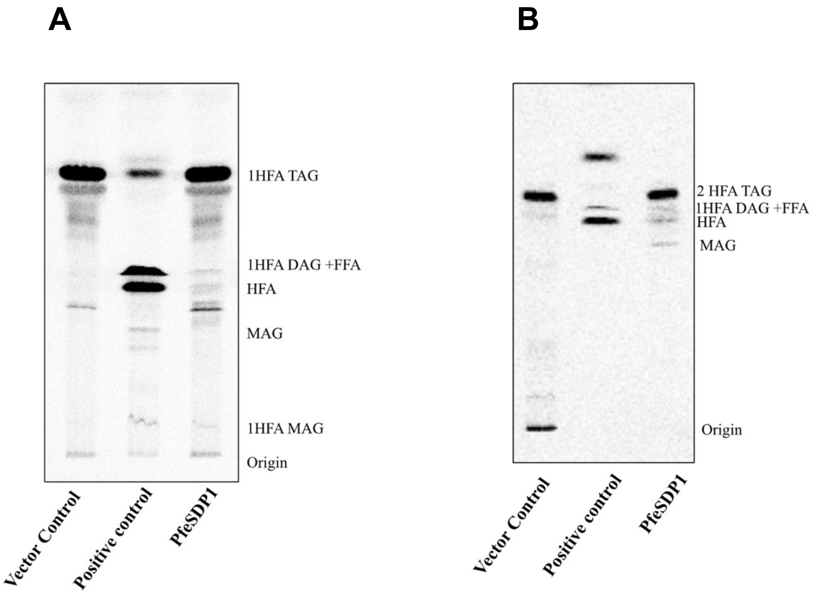

Supplementary Figure S2. In vitro lipase assay with [^{14}C]1HFA-TAG and [^{14}C]2HFA-TAG substrates. Phosphor image TLC of lipids extracted from *in vitro* lipase assay reactions with [^{14}C]1HFA-TAG **(A)** and [^{14}C]2HFA-TAG **(B)** as substrates. Lipase from *Rhizopus mehei* was used as positive control. Elution fraction from vector control (VC), was used as negative controls. 1HFA-TAG and 2HFA-TAG represents one and two hydroxy fatty acid containing triacylglycerols, respectively; 1HFA-DAG, one hydroxy fatty acid containing diacylglycerol; 1HFA-MAG, one hydroxy fatty acid containing monoacylglycerol; HFA, hydroxy fatty acid, FFA, free fatty acid; MAG, Monoacylglycerol.

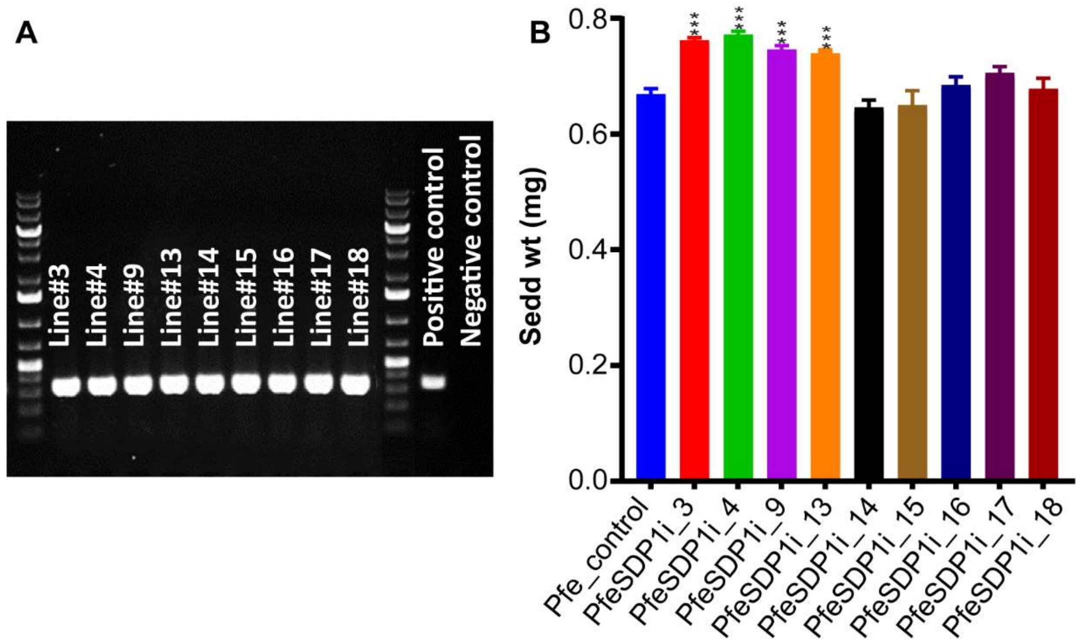

Figure S3. Transgenic PfeSDP1_RNAi line screening. (A) Transgenic PfeSDP1_RNAi plants were confirmed by PCR using 2S promoter and gene-specific primers, pB9-PfeSDP1 plasmid used as positive and DNA extraction buffer used as negative control. **(B)** Seed weight of T1 selfed plants (T2 seed) represents average seed weight (n=15). Asterisks (***) indicate significant differences at P≤0.0005 to the control by two-tailed paired *t*-tests.

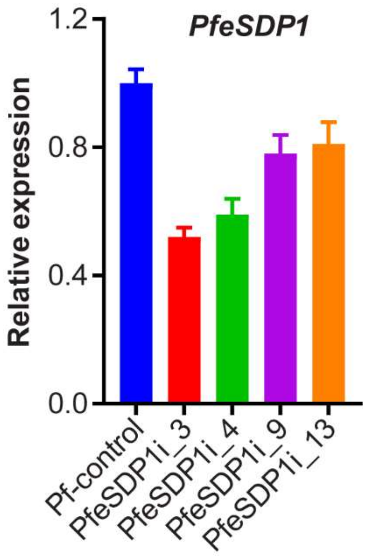

Supplementary Figure S4. **Relative expression of the *PfeSDP1* gene in PfeSDP1_RNAi lines.** Relative expression of the *PfeSDP1* gene in PfeSDP1_RNAi lines in T2 germinated seeds compared to the *P. fendleri* control germinated seeds. The gene expression was quantified by qRT-PCR and normalized to the reference *18S* gene. The expression values are the average of three biological replicates ± SE.

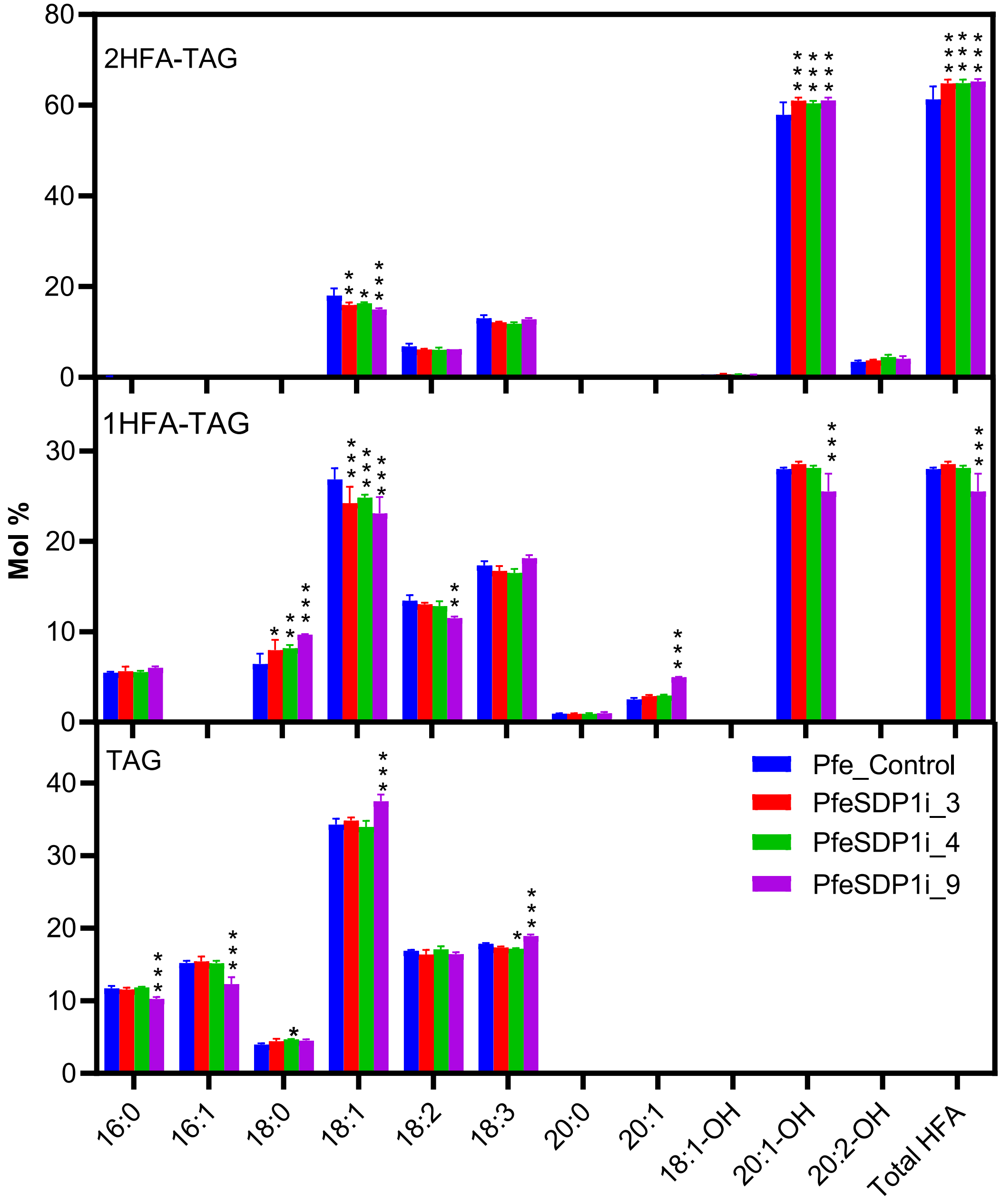

Supplementary Figure S5. Fatty acid profiles of different TAG molecular species in the seeds of PfeSDP1 knockdown lines. Fatty acid composition of TAG, 1HFA-TAG and 2HFA-TAG were represented as Mol % of total fatty acids. Values represent mean ± SD of three independent plants from each line. Significant difference between wild type control and PfeSDP1 knockdown lines was determined using two-way ANOVA (***P ≤ 0.001, **P≤ 0.002, *P≤ 0.033).

Supplementary Table S1. List of primers used in this study

Primer name	Sequence (5'-3)	Purpose
PfeSDP1-FW	ATATGGTACCCATGGATATAAGTAATGAGGCCAGT	Yeast Cloning
PfeSDP1-RV	ATATGCGGCCGCTCACTGAACAGAAACTGGTTCTT	Yeast Cloning
PfeSDP1-RNAiF	TACTACCGCGGCGGCCGCAATGGCTTACCGGAGGAAGT	RNAi
PfeSDP1-RNAiR	GACTTCTGCAGCCCGGGAAGTGAGCCCTCTTGACGAC	RNAi
2SProm-SeqFor2	CTTACACGTGATTGCCATGCAAATC	Squencing
F2I-5SEQR	CGAAAATGAGAAGAAATGGAAGAGAAGC	Squencing
F2I-3SEQF	CCATAGTCTTGAGTTTTCAGCTTGTTG	Squencing
GlycininTerm-SeqR2	CAGTGAACAAAAGGCAAGCTAAAGCC	Squencing
*Pfe18S*_F	GAGAAACGGCTACCACATCCA	qRT-PCR
*Pfe18S*_R	CCGTGTCAGGATTGGGTAATTT	qRT-PCR

Supplementary Table S2. Species abbreviations and accession numbers used in phylogenetic study of SDP1 proteins.

Accession	Species	Abbreviation
This study	*Physaria fendleri*	PfeSDP1
NP_001332745.1	*Arabidopsis thaliana*	AtSDP1
XP_010452294.1	*Camelina sativa*	CsaSDP1
XP_013668385.1	*Brassica napus*	BnaSDP1
XP_009122642.1	*Brassica rapa*	BraSDP1
XP_018446336.1	*Raphanus sativus*	RsSDP1
EEF32280.1	*Ricinus communis]*	RcSDP1
XP_015898084.1	*Ziziphus jujuba*	ZjSDP1
XP_007203312.1	*Prunus persica*	PpSDP1
XP_002308909.1	*Populus trichocarpa*	PtSDP1
XP_012085968.1	*Jatropha curcas*	JcSDP1
RVW92138.1	*Vitis vinifera*	VviSDP1
XP_039163397.1	*Eucalyptus grandis*	EgrSDP1

Supplementary Table S3. Fatty acid compositions (µg per mg seed) of *P. fendleri* expressing SDP1_RNAi

	**16:0**	**18:0**	**18:1**	**18:2**	**18:3**	**20:0**	**20:1**	**18:1-OH**	**18:2-OH**	**20:1-OH**	**20:2-OH**
**Pfe_control**	4.8±0.11	4.4±0.11	38.3±0.95	18.1±0.18	30.4±0.61	0.5±0.02	1.2±0.04	0.4±0.02	0.6±0.01	122.2±2.88	7.0±0.31
**PfeSDP1i_3**	4.8±0.05	4.4±0.19	44.2±0.85	19.8±0.45	31.6±1.27	0.4±0.01	1.5±0.03	0.3±0.01	0.7±0.02	144.8±2.08	8.9±0.44
**PfeSDP1i_4**	4.8±0.22	4.5±0.15	44.3±0.98	19.6±0.40	33.7±0.28	0.4±0.02	1.5±0.04	0.3±0.03	0.5±0.02	149.8±1.90	10.6±0.53
**PfeSDP1i_9**	4.2±0.14	4.1±0.15	41.6±0.78	19.6±0.36	35.9±0.76	0.3±0.01	1.8±0.06	0.2±0.03	0.3±0.01	153.6±1.42	9.8±0.13
**PfeSDP1i_13**	4.0±0.14	3.8±0.12	39.1±0.98	18.2±0.29	34.8±0.75	0.3±0.01	1.6±0.05	0.2±0.02	0.4±0.01	155.0±0.90	10.8±0.18

Supplementary Table: S4. Fatty acid compositions (weight percent of total FAs) of *P. fendleri* expressing SDP1_RNAi

**Pfe_control**	**16:0**	**18:0**	**18:1**	**18:2**	**18:3**	**20:0**	**20:1**	**18:1-OH**	**18:2-OH**	**20:1-OH**	**20:2-OH**
**Pfe_control**	2.1±0.12	2.0±0.11	16.8±0.20	8.0±0.20	13.3±0.13	0.2±0.01	0.6±0.01	0.2±0.01	0.3±0.01	53.5±0.37	3.1±0.12
**PfeSDP1i_3**	1.9±0.06	1.7±0.13	17.8±0.48	7.5±0.28	12.3±0.40	0.2±0.01	0.6±0.01	0.1±0.01	0.3±0.01	55.0±0.32	2.7±0.15
**PfeSDP1i_4**	1.8±0.11	1.7±0.07	16.4±0.49	7.3±0.17	12.5±0.10	0.2±0.01	0.6±0.01	0.1±0.01	0.2±0.01	55.4±0.45	3.9±0.18
**PfeSDP1i_9**	1.5±0.05	1.5±0.05	15.3±0.16	7.2±0.07	13.2±0.17	0.1±0.00	0.7±0.02	0.1±0.01	0.1±0.00	56.5±0.08	3.6±0.01
**PfeSDP1i_13**	1.5±0.06	1.4±0.04	14.5±0.25	6.8±0.09	13.0±0.23	0.1±0.00	0.6±0.02	0.1±0.01	0.1±0.00	57.7±0.25	4.1±0.08
